# Prescription patterns of hypnotics for children and adolescents in Japan: a descriptive epidemiologic study using a claims database

**DOI:** 10.1093/sleep/zsag067

**Published:** 2026-03-09

**Authors:** Sachiko Tanaka-Mizuno, Kenichi Fujimoto, Kazuo Mishima, Yukinori Sakata, Motomu Suga, Hiroshi Ohashi, Naoki Kubota, Michinori Koebis, Toshiki Fukasawa, Kayoko Mizuno, Mika Ishii, Margaret Moline, Koji Kawakami

**Affiliations:** Laboratory of Epidemiology and Prevention, Kobe Pharmaceutical University, Kobe, Japan; Department of Pharmacoepidemiology, Graduate School of Medicine and Public Health, Kyoto University, Kyoto, Japan; Eisai Co., Ltd., Tokyo, Japan; Department of Neuropsychiatry, Akita University Graduate School of Medicine, Akita, Japan; Eisai Co., Ltd., Tokyo, Japan; Graduate School of Clinical Psychology, Teikyo Heisei University, Tokyo, Japan; National Hospital Organization Mie National Hospital, Mie, Japan; Eisai Co., Ltd., Tokyo, Japan; Eisai Co., Ltd., Tokyo, Japan; Department of Pharmacoepidemiology, Graduate School of Medicine and Public Health, Kyoto University, Kyoto, Japan; Department of Digital Health and Epidemiology, Graduate School of Medicine and Public Health, Kyoto University, Kyoto, Japan; Department of Pharmacoepidemiology, Graduate School of Medicine and Public Health, Kyoto University, Kyoto, Japan; Department of Digital Health and Epidemiology, Graduate School of Medicine and Public Health, Kyoto University, Kyoto, Japan; Eisai Co., Ltd., Tokyo, Japan; Eisai Inc., NJ, United States; Department of Pharmacoepidemiology, Graduate School of Medicine and Public Health, Kyoto University, Kyoto, Japan

**Keywords:** adolescents, children, claims database, hypnotics, prescription, patterns

## Abstract

**Study Objectives:**

To investigate the prescription patterns for hypnotics among patients <18 years in Japan.

**Methods:**

We conducted a descriptive epidemiologic study using a claims database in Japan. We included patients aged 0–17 years with first-time prescriptions for hypnotics between June 2021 and June 2023. Hypnotics were classified as melatonin, melatonin receptor agonists (MRAs), dual orexin receptor antagonists (DORAs), Z-drugs, and benzodiazepines. We described the types of hypnotics, dosages, prescription duration, comorbidities, and concomitant medications for overall and by age groups.

**Results:**

The study included 21 145 patients; 1983 aged 0–6 years, 3901 aged 7–11 years, 6664 aged 12–14 years, and 8597 aged 15–17 years. The initial prescribed hypnotics were melatonin (33.9%), MRAs (30.9%), DORAs (18.8%), Z-drugs (10.1%), and benzodiazepines (6.3%). Melatonin was more likely to be prescribed to patients age 0–11 years, MRAs to those aged 0–17 years, and DORAs to patients ages 15–17 years. The most common mental health-related comorbidities were autism spectrum disorder (32.5%), depression (23.4%), attention-deficit hyperactivity disorder (19.8%), and anxiety disorder (18.2%).

**Conclusions:**

Melatonin was most frequently prescribed in children for a longer duration than other hypnotics, while MRAs were prescribed across all age groups and DORAs mainly to adolescents. These age-specific patterns suggest that the drug selection was conducted according to patient’s age, since pediatric insomnia might be correlated with psychiatric disorders related to developmental stage. Future research should investigate the long-term effects of hypnotic prescriptions with consideration of comorbid conditions.

## Graphical Abstract

**Graphical Abstract:**
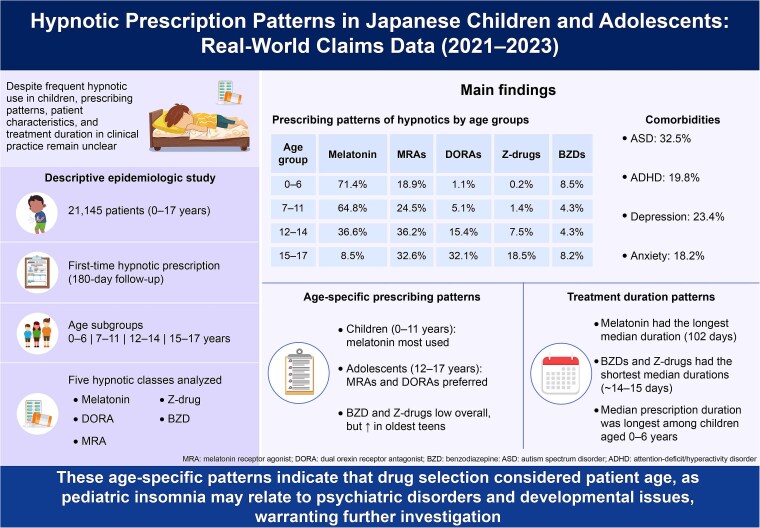

Statement of Significance This is the first study to describe hypnotics use in Japanese children and adolescents using a large-scale claims database. It reveals age-specific prescription patterns—melatonin for children and dual orexin receptor antagonists for adolescents—reflecting tailored clinical decisions. These patterns are influenced by comorbidities, with autism spectrum disorder and attention-deficit/hyperactivity disorder being prevalent in children ages 0–11 years, and depressive and anxiety disorders being more common in adolescents ages 12–17 years. The findings fill a critical gap in pediatric sleep pharmacotherapy and highlight the need for further research into long-term safety and efficacy, especially in relation to neurodevelopmental and mental health outcomes.

## Introduction

Insomnia is a clinical and public health concern among children and adolescents. Previous studies in Japan have reported that 18%–24% of children and adolescents experience sleep disorders [1–3]. A prominent feature of pediatric insomnia is its strong association with neurodevelopmental disorders, such as autism spectrum disorder (ASD) and attention-deficit/hyperactivity disorder (ADHD), and psychiatric conditions such as depression and anxiety disorders [4–6]. Therefore, a comprehensive care approach that considers psychiatric comorbidities is required for insomnia treatment in children [5]. Since insomnia in children may present with different clinical characteristics than those in adults, adult treatment strategies cannot necessarily be applied directly to pediatric patients. Additionally, persistent insomnia during childhood increases the risk of adverse effects on physical and mental development, mood and behavioral regulation, and academic performance; consequently, appropriately managing pediatric insomnia would potentially mitigate a significant public health problem in the future [1, 7].

Although non-pharmacological therapy is recommended as the first-line treatment for adult insomnia international clinical practice guideline [8], pharmacotherapy remains the mainstay of treatment in clinical practice. For adult patients, four classes of hypnotics are mainly used for insomnia treatment: benzodiazepines (BZDs), Z-drugs, melatonin receptor agonists (MRAs), and dual orexin receptor antagonists (DORAs). In contrast, the treatment options for pediatric patients are more limited. Melatonin was launched in June 2020 in Japan for the treatment of sleep-onset difficulties in children with neurodevelopmental disorders and is currently the only medication with an approved pediatric indication. Melatonin is widely used internationally as a treatment for pediatric insomnia, generally as an over-the-counter supplement. For instance, the American Academy of Neurology recommends that clinicians offer melatonin to children and adolescents with ASD if behavioral therapy is not helpful [9]. A formulation of melatonin is approved in the EU/UK for prescription use. European experts suggest offering melatonin to typically developing children only after sleep hygiene and behavioral interventions have been ineffective [10]. Hypnotics other than melatonin are therefore used off-label in pediatric patients. Although previous studies have evaluated insomnia medication use in children and adolescents, they were either reports from outside Japan or based on data before the approval of melatonin in Japan [11–14]. Therefore, the current real-world prescribing patterns in Japan remain unclear. Among adult patients in Japan, there has been an increasing switch from BZDs to DORAs, as well as prescribing DORAs to newly diagnosed patients [15–17]. Given these changes in adult practice and the limited evidence in pediatric patients, it is crucial to investigate the actual prescription practice to establish a therapeutic algorithm and ensure appropriate management for pediatric insomnia.

Therefore, we conducted a descriptive epidemiologic study using data from a large-scale claims database to clarify the patterns of hypnotic prescriptions in clinical practice among children and adolescents in Japan. We described the characteristics of patients receiving these prescriptions, including comorbidities and concomitant medications, and analyzed the types of medications prescribed, initial doses, and duration of treatment. In this study, we aimed to provide foundational data on the clinical practice of insomnia treatment among children and adolescents to fill the knowledge gap in the context of frequent off-label use.

## Materials and methods

### Data source and setting

We used a claims database provided by JMDC Inc. (Tokyo, Japan) [18, 19]. It is a large-scale database of health insurance claims that aggregates inpatient and outpatient diagnoses, procedures, and pharmacy dispensing records since January 2005. As of November 2025, the cumulative dataset includes approximately 22 million insured individuals and their dependents. The proportion of individuals aged 0–17 years accounted for approximately 20% of the total population in this database. An encrypted personal identifier enables linkage of claims across hospitals and clinics, except when beneficiaries switch insurers, allowing longitudinal tracking within the same insurance system. Due to its scale and coverage, the claims database from JMDC Inc. has been widely used for epidemiologic studies in Japan [16, 20]. In this study, we used information from this claims database for the period from January 2005 to December 2023.

### Study design and patients

We conducted a descriptive epidemiologic study using the claims database to investigate the prescription patterns for hypnotics among children and adolescents in Japan. We initially identified patients aged 0–17 years who were prescribed hypnotics for the first time between June 2021 and June 2023. We chose June 2021 as the start date, corresponding to when the restriction on the prescription period for melatonin, the only hypnotic with a pediatric indication, was lifted, and June 2023 as the end date to ensure a 180-day follow-up period. We selected 16 hypnotics based on the Anatomical Therapeutic Chemical (ATC) codes listed in Table S1 all these medications have an approved indication for insomnia in Japan. We defined the first prescription date of hypnotics as the index date (Day [0]). Inclusion criteria were as follows: (1) continuous database enrollment for at least 180 days before the index date (baseline period), (2) no prescription of any hypnotics during the baseline period, and (3) continuous enrollment for at least 180 days after the index date (follow-up period).

### Variables: prescription of hypnotics

We included the following hypnotics used in Japan: melatonin, BZDs (estazolam, flurazepam, nitrazepam, triazolam, flunitrazepam, brotizolam, lormetazepam, quazepam, rilmazafone), Z-drugs (zopiclone, zolpidem, eszopiclone), MRAs (ramelteon), and DORAs (suvorexant and lemborexant). We categorized hypnotics prescribed on the index date into these five pharmacological classes. Definitions of these medications are detailed in Table S1. Although clinicians sometimes use antipsychotics and antidepressants for insomnia treatment [21–23], these drugs are often prescribed for other indications, such as ASD or depression, and claims data do not clearly distinguish whether they are used for insomnia or for other symptoms; therefore, we did not include these drugs in the primary analysis of this study, and focused only hypnotics.

We measured the details of prescription patterns for first-time hypnotics use at the index date. We also classified prescription patterns into two patterns, monotherapy and polytherapy, with monotherapy defined as the prescription of one hypnotic and polytherapy as the prescription of two or more hypnotics prescribed simultaneously on the index date. Dosage at the index date was transformed to a flunitrazepam-equivalent (FZE) dose (FZE eq. mg/day) and the original dose [24]. For example, it can be posited that 4 mg/day of melatonin corresponds to 1 mg of FZE. The conversion factors used to convert doses to FZE are shown in Table S1.

We also evaluated the prescription continuation period for the initially prescribed hypnotics. We calculated the period during which prescriptions for the index hypnotic were continued without a gap of more than 28 days between two consecutive prescriptions and defined the prescription period as the shorter of this period and the 180-day follow-up period.

### Other variables

We specified a baseline period from −180 to 0 days prior to the index date and a follow-up period from 1 to 180 days after the index date. Disease names and drugs were defined using the International Classification of Diseases, Tenth Edition (ICD-10) codes, and ATC codes. We described baseline characteristics and mental health-related comorbidities at baseline. Mental health-related comorbidities included any mental disorder, schizophrenia and psychiatric disorder, depression, bipolar affective disorder, anxiety disorder, post-traumatic stress disorder, insomnia, intellectual disability, ASD, ADHD, and tic disorder. Other comorbidities included diabetes, obstructive sleep apnea, restless legs syndrome, food allergy, anaphylactic shock, allergic rhinitis, asthma, and atopic dermatitis. We also described the psychotropic drugs other than hypnotics, including antipsychotics, antiepileptics, antidepressants, anxiolytics, and ADHD drugs. We summarized medication history during the baseline period (excluding the index date) and concomitant medications on the index date separately. The details of ICD-10 codes and ATC codes are listed in Table S2.

We categorized the age subgroups as: 0–6 years, 7–11 years, 12–14 years, and 15-17 years. Age groups were defined based on the Japanese school education system: 0–6 years, 7–11 years, 12–14 years, and 15–17 years.

### Statistical analysis

Descriptive statistics were used to summarize baseline and clinical characteristics. Categorical variables were expressed as frequencies and percentages, and continuous variables were presented as means and standard deviations (SDs) or medians and interquartile ranges (IQRs).

We first examined the initially prescribed hypnotics and described the proportions of monotherapy and polytherapy, as well as the prescribing proportions of each medication class and individual agent. We then examined the prescription patterns and described the initial daily doses and the duration of continuous treatment. For the initial daily doses, both the original dose (mg/day) and the FZE dose were used. We examined the prescription patterns only for patients whose initial hypnotic was monotherapy (excluding polytherapy on the index date).

Additionally, to assess evolving treatment patterns, the proportion of patients with concomitant psychotropic prescriptions were reported at several intervals during a 180-day follow-up period.

We examined prescriptions of the initial hypnotic and any additional hypnotics after the index date. For each of the 16 hypnotics, we assessed whether it was prescribed on days 0, 30, 60, 90, 120, 150 and 180 from the index date. Prescriptions were summarized for each hypnotic and for each drug class, and cases with prescriptions from two or more hypnotic classes were categorized as polytherapy.

We conducted all analyses for all patients, as well as stratified them by age group and by pharmacological class. All statistical analyses were performed using SAS version 9.4 (SAS Institute Inc., Cary, NC, USA).

## Results

We identified 3 349 453 individuals aged 0–17 years during the study period from June 2021 to June 2023. Of these, 29 096 patients received at least one prescription for a hypnotic. After applying the exclusion criteria, 21 145 patients were included in the final cohort (Figure S1).

The baseline characteristics of the study cohort are presented in Table 1**.** The mean age was 13.3 years (SD, 3.86), and 53.6% of the patients were girls. The proportion of girls increased with age, rising from 36.0% in the 0–6 years age group to 60.3% in the 15–17 years age group. During the baseline period, the most frequently recorded mental-related comorbidities were insomnia (67.6%), ASD (32.5%), depression (23.4%), ADHD (19.8%), and anxiety disorders (18.2%). Other prevalent conditions included allergic rhinitis (40.8%), asthma (20.0%), and atopic dermatitis (14.1%). Since the proportion of patients with a recorded history of insomnia (67.6%) was based on diagnosis records in claims data, it may not accurately reflect the true prevalence of insomnia among the patients. The prevalence of these conditions varied by age; ASD and ADHD were most common in younger patients (55.6% and 41.8%, respectively, in the 7–11 years age group), whereas depression and anxiety disorders were most prevalent in the 15–17 years age group (37.2% and 24.4%, respectively). Regarding prior psychotropic use, 19.9% of the cohort had received prescriptions for antipsychotics, 10.4% for antidepressants, and 12.9% for anxiolytics. The use of antipsychotics and ADHD drugs was most frequent in the 7–11 year age group (26.6% and 28.1%, respectively).

**Table 1:** Baseline and clinical characteristics in children and adolescents who initiated hypnotics in Japan

			Overall		0–6 years		7–11 years		12–14 years		15–17 years	
			N = 21 145		N = 1983		N = 3901		N = 6664		N = 8597	
			n	%	n	%	n	%	n	%	n	%
Sex, girl			11 339	53.6	713	36.0	1572	40.3	3870	58.1	5184	60.3
Age (years old), mean and SD			13.3	3.86	4.52	1.61	9.86	1.41	13.71	0.82	16.56	0.86
Comorbidities in the 180 days up to and including the index date (days −180 to 0)												
	Insomnia		14 292	67.6	796	40.1	2022	51.8	4551	68.3	6923	80.5
	Allergic rhinitis		8634	40.8	1084	54.7	2119	54.3	2783	41.8	2648	30.8
	ASD		6869	32.5	1114	56.2	2169	55.6	2178	32.7	1408	16.4
	Depression		4938	23.4	4	0.2	249	6.4	1484	22.3	3201	37.2
	Asthma		4227	20.0	863	43.5	1233	31.6	1226	18.4	905	10.5
	ADHD		4185	19.8	297	15.0	1632	41.8	1306	19.6	950	11.1
	Anxiety disorder		3853	18.2	93	4.7	447	11.5	1214	18.2	2099	24.4
	Atopic dermatitis		2982	14.1	507	25.6	658	16.9	874	13.1	943	11.0
	Schizophrenia and psychotropic-related disease		2791	13.2	64	3.2	378	9.7	904	13.6	1445	16.8
	Epilepsy		1500	7.1	299	15.1	375	9.6	375	5.6	451	5.2
	Intellectual disability		1442	6.8	427	21.5	426	10.9	327	4.9	262	3.0
	Bipolar disorder		1042	4.9	11	0.6	98	2.5	287	4.3	646	7.5
	Food allergy		657	3.1	144	7.3	165	4.2	207	3.1	141	1.6
	Circadian rhythm sleep disorders		468	2.2	42	2.1	89	2.3	190	2.9	147	1.7
	Diabetes		299	1.4	8	0.4	29	0.7	75	1.1	187	2.2
	Tic disorder		151	0.7	11	0.6	68	1.7	50	0.8	22	0.3
	Anaphylactic Shock		135	0.6	18	0.9	24	0.6	49	0.7	44	0.5
	PTSD		120	0.6	1	0.1	17	0.4	39	0.6	63	0.7
	Obstructive sleep apnea		119	0.6	24	1.2	22	0.6	36	0.5	37	0.4
	Restless legs syndrome		57	0.3	6	0.3	19	0.5	15	0.2	17	0.2
	Orthostatic hypotension		16	0.1	0	0.0	10	0.3	3	0.0	3	0.0
Drug history of psychotropic drugs in the 180 days before the index date (days −180 to −1)												
	Antipsychotics		4205	19.9	239	12.1	1037	26.6	1311	19.7	1618	18.8
	Antidepressants		2196	10.4	4	0.2	165	4.2	662	9.9	1365	15.9
	Anxiolytics		2736	12.9	203	10.2	234	6.0	704	10.6	1595	18.6
	ADHD drugs		2387	11.3	84	4.2	1098	28.1	737	11.1	468	5.4
	Antiepileptics		1597	7.6	270	13.6	339	8.7	413	6.2	575	6.7
Concomitant psychotropic drugs at the index date (day 0)												
	Antipsychotics		4005	18.9	222	11.2	933	23.9	1237	18.6	1613	18.8
	Antidepressants		2234	10.6	2	0.1	127	3.3	612	9.2	1493	17.4
	Anxiolytics		2074	9.8	93	4.7	132	3.4	495	7.4	1354	15.7
	ADHD drugs		2018	9.5	64	3.2	968	24.8	596	8.9	390	4.5
	Antiepileptics		1060	5.0	182	9.2	222	5.7	260	3.9	396	4.6

ADHD, Attention-Deficit/Hyperactivity Disorder; ASD: Autism Spectrum Disorder; PTSD, Post-Traumatic Stress Disorder; SD, Standard Deviation

Table 2 shows the initial prescription patterns of hypnotics. Most patients (98.1%) started treatment with a monotherapy. The overall proportion of polytherapy was low (1.9%) but increased with age from 0.5% in the 0–6 years age group to 3.0% in the 15–17 years age group. The most common pharmacological classes for patients receiving monotherapy were melatonin (33.9%), MRAs (30.9%), and DORAs (18.8%). Prescription patterns varied according to age. Melatonin was the most common choice for younger children, accounting for 71.4% of prescriptions in the 0–6 years age group and 64.8% in the 7–11 years age group. In contrast, MRAs and DORAs were the most frequent choices for adolescents, comprising 51.6% of the prescriptions in the 12–14 years age group and 64.7% in the 15–17 years age group. BZDs (6.3%) and Z-drugs (10.1%) were overall less common but were used more often in the oldest adolescents. The three most prescribed hypnotics were melatonin (33.9%), ramelteon (30.9%), and lemborexant (14.6%). Patient characteristics also differed according to the pharmacological class of the initiated hypnotics (Table S3).

**Table 2:** Prescribing patterns of hypnotics among children and adolescents in Japan

		Overall		0–6 years		7–11 years		12–14 years		15–17 years	
		N = 21 145		N = 1983		N = 3901		N = 6664		N = 8597	
Hypnotics: monotherapy vs. polytherapy											
		n	%	n	%	n	%	n	%	n	%
Monotherapy		20 736	98.1	1974	99.5	3870	99.2	6552	98.3	8340	97.0
Polytherapy		409	1.9	9	0.5	31	0.8	112	1.7	257	3.0
Hypnotics by pharmacological class (monotherapy)											
		n	%	n	%	n	%	n	%	n	%
Melatonin		7025	33.9	1409	71.4	2506	64.8	2398	36.6	712	8.5
BZDs		1297	6.3	167	8.5	165	4.3	282	4.3	683	8.2
Z-drugs		2096	10.1	4	0.2	56	1.4	490	7.5	1546	18.5
MRAs		6411	30.9	373	18.9	947	24.5	2373	36.2	2718	32.6
DORAs		3907	18.8	21	1.1	196	5.1	1009	15.4	2681	32.1
Hypnotics drugs (monotherapy)											
		n	%	n	%	n	%	n	%	n	%
Melatonin		7025	33.9	1409	71.4	2506	64.8	2398	36.6	712	8.5
BZDs	Brotizolam	568	2.7	8	0.4	28	0.7	143	2.2	389	4.7
	Nitrazepam	307	1.5	105	5.3	78	2.0	46	0.7	78	0.9
	Triazolam	173	0.8	2	0.1	15	0.4	45	0.7	111	1.3
	Estazolam	121	0.6	48	2.4	33	0.9	15	0.2	25	0.3
	Flunitrazepam	60	0.3	3	0.2	6	0.2	18	0.3	33	0.4
	Lormetazepam	52	0.3	0	0.0	2	0.1	12	0.2	38	0.5
	Rilmazafone	68	0.3	0	0.0	4	0.1	18	0.3	46	0.6
	Flurazepam	8	0.0	1	0.1	3	0.1	1	0.0	3	0.0
	Quazepam	8	0.0	0	0.0	0	0.0	2	0.0	6	0.1
Z-Drugs	Zolpidem	1143	5.5	3	0.2	37	1.0	286	4.4	817	9.8
	Eszopiclone	839	4.0	0	0.0	15	0.4	178	2.7	646	7.7
	Zopiclone	46	0.2	1	0.1	0	0.0	8	0.1	37	0.4
MRAs	Ramelteon	6411	30.9	373	18.9	947	24.5	2373	36.2	2718	32.6
DORAs	Lemborexant	3021	14.6	10	0.5	139	3.6	775	11.8	2097	25.1
	Suvorexant	886	4.3	11	0.6	57	1.5	234	3.6	584	7.0

BZDs, Benzodiazepines; DORAs, Dual Orexin Receptor Antagonists; MRAs, Melatonin Receptor Agonists.

Table 3 presents the initial daily doses of hypnotics. The mean FZE dose for all hypnotics was 0.53 mg/day (±0.31), and this standardized dose increased with age, from 0.29 mg/day in the 0–6 years age group to 0.64 mg/day in the 15–17 years age group. By pharmacological class, BZDs had the highest mean FZE dose (0.89 mg/day), whereas melatonin had the lowest (0.29 mg/day).

**Table 3:** Initial prescription dose of hypnotics: overall and by age group

	Overall			0–6 years			7–11 years			12–14 years			15–17 years		
	N	Mean	SD	N	Mean	SD	N	Mean	SD	N	Mean	SD	N	Mean	SD
All hypnotics, flunitrazepam-equivalent dose (mg FZE/day^a^)															
	20 736	0.53	0.31	1974	0.29	0.21	3870	0.40	0.26	6552	0.54	0.31	8340	0.64	0.31
Hypnotics class, flunitrazepam-equivalent dose (mg FZE/day^a^)															
Melatonin	7025	0.29	0.14	1409	0.24	0.14	2506	0.29	0.13	2398	0.32	0.15	712	0.33	0.16
BZDs	1297	0.89	0.34	167	0.58	0.34	165	0.79	0.36	282	0.94	0.29	683	0.96	0.31
Z-drugs	2096	0.57	0.21	4	0.64	0.48	56	0.47	0.16	490	0.53	0.20	1546	0.58	0.22
MRAs	6411	0.75	0.30	373	0.34	0.22	947	0.63	0.29	2373	0.77	0.29	2718	0.84	0.27
DORAs	3907	0.44	0.21	21	0.24	0.12	196	0.36	0.19	1009	0.41	0.20	2681	0.46	0.21
Top 8 most prescribed hypnotics, original dose^b^ (mg/day)															
Melatonin	7025	1.17	0.57	1409	0.97	0.54	2506	1.16	0.52	2398	1.26	0.59	712	1.30	0.64
Ramelteon	6411	6.01	2.42	373	2.74	1.74	947	5.03	2.31	2373	6.14	2.29	2718	6.69	2.16
Lemborexant	3021	3.68	1.47	10	1.98	0.69	139	2.89	1.44	775	3.40	1.36	2097	3.85	1.47
Zolpidem	1143	5.51	1.79	0	-	-	37	4.66	1.34	286	5.12	1.57	817	5.69	1.84
Suvorexant	886	13.97	3.87	11	5.48	3.07	57	10.41	3.84	234	13.26	3.57	584	14.76	3.55
Eszopiclone	839	1.39	0.56	3	4.13	1.50	15	1.03	0.30	178	1.35	0.57	646	1.41	0.56
Brotizolam	568	0.24	0.04	8	0.25	0.00	28	0.21	0.08	143	0.24	0.05	389	0.25	0.03
Nitrazepam	307	3.51	2.08	105	2.31	1.73	78	3.78	2.17	46	4.37	1.60	78	4.34	1.94

a Daily doses of insomnia medications were transformed by flunitrazepam-equivalent conversion.

b Original dose indicates the dosage standards of pharmaceuticals approved in Japan.

BZDs, Benzodiazepines; DORAs, Dual Orexin Receptor Antagonists; FZE, flunitrazepam-equivalent; MRAs, Melatonin Receptor Agonists; SD, standard deviation.

On their original dose, the three most prescribed medications—melatonin, ramelteon, and lemborexant—had mean initial daily doses of 1.17, 6.01, and 3.68 mg, respectively, and for all three, the initial dose increased with age. For melatonin, the mean dose aligned with the labeled pediatric starting dose (1 mg), and remained below the 4 mg maximum. For ramelteon and lemborexant, despite the absence of pediatric reference standards, the observed doses were lower than those typically used in adults, with the most common adult doses of 8 mg and 5 mg, respectively.

Table 4 summarizes the prescription duration of hypnotics. For all patients, the median prescription duration for hypnotics was 44 days (IQR, 14–159 days). The median prescription duration was longest among children aged 0–6 years (114 days) and shortest among adolescents aged 15–17 years (30 days). By pharmacological class of initial hypnotic, patients prescribed melatonin had the longest median duration of 102 days (IQR, 30–180 days). In contrast, those prescribed BZDs and Z-drugs had the shortest median durations, with 14 and 15 days, respectively.

**Table 4:** Prescription period after the initiation of hypnotics: overall and by age group

		Overall				0–6 years				7–11 years				12–14 years				15–17 years			
All hypnotics, Days																					
		N	median	Q1	Q3	N	median	Q1	Q3	N	median	Q1	Q3	N	median	Q1	Q3	N	median	Q1	Q3
		20 736	44	14	159	1974	114	21	180	3870	90	28	180	6552	50	20	147	8340	30	14	87
Hypnotics by pharmacological class, Days																					
		N	median	Q1	Q3	N	median	Q1	Q3	N	median	Q1	Q3	N	median	Q1	Q3	N	median	Q1	Q3
	Melatonin	7025	102	30	180	1409	180	35	180	2506	130	30	180	2398	80	30	180	712	58	28	168
	BZDs	1297	14	1	51	167	1	1	21	165	1	1	25	282	14	5	50	683	21	7	63
	Z-drugs	2096	15	7	53	4	1	1	1	56	14	5	68	490	14	7	50	1546	18	8	53
	MRAs	6411	43	15	127	373	48	7	180	947	59	14	180	2373	51	21	133	2718	35	14	98
	DORAs	3907	30	14	93	21	35	20	180	196	56	15	180	1009	35	14	106	2681	30	14	85
Top 8 most prescribed hypnotics, Days																					
		N	median	Q1	Q3	N	median	Q1	Q3	N	median	Q1	Q3	N	median	Q1	Q3	N	median	Q1	Q3
	Melatonin	7025	102	30	180	1409	180	35	180	2506	130	30	180	2398	80	30	180	712	58	28	168
	Ramelteon	6411	43	15	127	373	48	7	180	947	59	14	180	2373	51	21	133	2718	35	14	98
	Lemborexant	3021	33	14	100	10	30	16	180	139	52	14	180	775	36	14	119	2097	30	14	93
	Zolpidem	1143	14	7	48	3	1	1	1	37	10	5	56	286	14	7	47	817	15	8	49
	Suvorexant	886	28	10	73	11	81	20	180	57	92	28	180	234	30	14	84	584	21	10	58
	Eszopiclone	839	20	10	59	0	-	-	-	15	28	5	84	178	15	10	58	646	21	10	58
	Brotizolam	568	20	7	58	8	1	1	1	28	4	1	14	143	21	7	57	389	21	9	63
	Nitrazepam	307	5	1	45	105	1	1	63	78	2	1	39	46	10	1	35	78	10	1	32

BZDs, Benzodiazepines; DORAs, Dual Orexin Receptor Antagonists; MRAs, Melatonin Receptor Agonists; Q1, 25th percentile; Q3, 75th percentile; SD, standard deviation.

**Figure 1:**
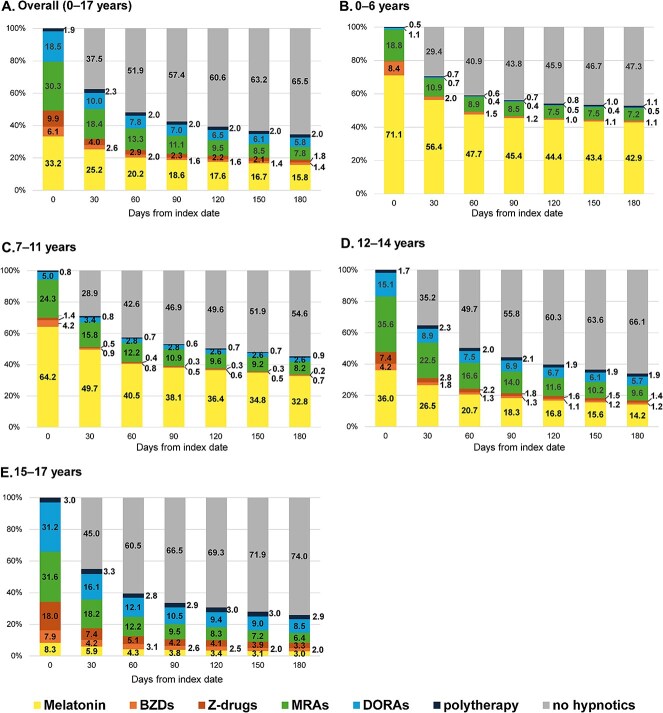
Longitudinal changes in insomnia medication patterns overall and by age group stacked bar charts show the proportions of patients at each time point (0, 30, 60, 90, 120, 150, and 180 days after the index date) who were receiving melatonin, benzodiazepines (BZDs), Z-drugs, melatonin receptor agonists (MRAs), dual orexin receptor antagonists (DORAs), more than two classes of hypnotics (≥2 hypnotic classes), or no hypnotic drug. Panels show (A) overall (0–17 years), (B) 0–6 years, (C) 7–11 years, (D) 12–14 years, and (E) 15–17 years.

Figure 1 illustrates the longitudinal changes in prescription patterns over the follow-up period. For all patients (Figure 1A), the proportion of patients prescribed hypnotics decreased over time, whereas the proportion of patients who discontinued hypnotics increased from 0% on day 0 to 65.5% on day 180. These patterns indicate clear age-related differences. In the younger age groups (0–6 and 7–11 years, Figure 1, B and C), the proportions of patients who had discontinued hypnotics by day 180 were 47.3% and 54.6%, respectively, which were lower than those in the older age groups. In addition, melatonin remained the most prescribed hypnotic throughout the follow-up period, accounting for 71.1% and 42.9% of prescriptions on days 0 and 180 in 0–6 years group, and 64.2% and 32.8% on days 0 and 180 in 7–11 years group, respectively. In contrast, for adolescents aged 12–17 years, the treatment patterns differ from those in children. In the 15–17 years group (Figure 1, E), while MRAs and DORAs were the most prescribed hypnotics on day 0 (31.6% and 31.2%, respectively), 74.0% of patients discontinued hypnotics at day 180. Figure S2 shows the longitudinal prescription patterns of the most prescribed hypnotics. Among patients who initiated melatonin, 52.6% had discontinued all hypnotics by day 180. Among those who started ramelteon or lemborexant, 68.4% and 71.3%, respectively, discontinued all hypnotics by day 180. Switching to or adding other hypnotics was rare in all initial treatment groups.

## Discussion

To the best of our knowledge, this is the first descriptive study to investigate the clinical practice of prescribing hypnotics to children (<12 years) and adolescents (12–17 years) in Japan. This study yielded two primary findings. First, the choice of hypnotics was clearly stratified by age; melatonin was the most common drug prescribed for children, whereas MRAs and DORAs were increasingly prescribed to adolescents. Second, patient comorbidities were also distinctly stratified by age, with neurodevelopmental disorders, such as ASD and ADHD, being prevalent in younger patients aged 0–14 years, and depressive and anxiety disorders being more common in older patients aged 12–17 years. These findings suggest that clinical practice for the pharmacological treatment of pediatric insomnia in Japan involves individualized care tailored to the patients’ age, developmental stage, and comorbidities.

### Prescribing patterns: choice of medications

In this study, melatonin was the most frequently prescribed agent, accounting for 33.9% of all hypnotic prescriptions, and was particularly common in the 0–14 years age group. This is likely attributable to the fact that melatonin is the only medication indicated for pediatric insomnia in Japan and is widely recognized for its favorable safety profile. The widespread use of melatonin is consistent with prescription trends in other countries, such as Sweden and Denmark [25, 26]. In contrast, our findings revealed that melatonin was widely used in age groups outside the covered range of 6–15 years. This off-label use by age may be clinically supported by international trials reporting on the efficacy and safety of melatonin in populations that include patients <6 and >15 years old [27, 28]; however, these studies were based on relatively small cohorts, and reports from Asian populations are still lacking. Therefore, future studies should evaluate the generalizability of these findings to the Japanese pediatric population.

Although the high prescription proportion of melatonin is consistent with international trends, the frequent use of MRAs and DORAs reveals a unique prescription pattern pertaining to Japan. Following melatonin, the most frequently prescribed medications were MRA ramelteon (30.9%) and DORA lemborexant (14.6%), with these three agents comprising approximately 80% of all prescriptions. Ramelteon had a high prescription rate across all age groups, potentially because it was used as a relatively safe agent for children before the approval of melatonin in 2019, a prescription practice that may have continued to date. There was a high proportion of lemborexant prescription among patients aged 15–17 years. This can likely be attributed to the Japanese clinical guidelines that recommend lemborexant for adults, and the official package insert defining an adult as one aged ≥15 years, leading to a similar therapeutic approach for older adolescents [29]. The prescription patterns for these medications differ from those in international practice; studies in the US and China report that MRAs and DORAs are rarely prescribed to children and adolescents with insomnia [22, 23]. In addition to the limited evidence from overseas, most clinical trials in Japan have been conducted in adults aged >18 years, underscoring the urgent need to establish domestic evidence on the long-term efficacy of these medications in younger populations.

The frequent prescription of melatonin and ramelteon in younger children and lemborexant in adolescents likely reflects not only expectations of efficacy but also safety considerations. In younger children, melatonin is often perceived to have a lower risk of oversedation and dependence compared with traditional hypnotics [30], and its granule formulation may facilitate dose adjustment, particularly in children with lower body weight. For adolescents, lemborexant may be selected based on its relatively favorable safety profile in adults [29]. These patterns suggest that clinicians prioritize the risk–benefit balance when selecting hypnotics for pediatric patients with insomnia. Given that the robust pediatric evidence for both efficacy and safety remain limited, further research is needed to support these clinical decisions.

Melatonin and ramelteon may be used to treat circadian rhythm disorders [31, 32]. In Table S3, approximately 3% of patients who initiated melatonin or ramelteon had a history of circadian rhythm disorders, which was higher than the proportion observed for other hypnotics. Since the claims database does not capture the clinical intent for prescribing, it remains unclear to what extent these medications were used for circadian rhythm disorders versus insomnia or other sleep disorders. Nevertheless, melatonin is indicated in Japan for “difficulty falling asleep associated with neurodevelopmental disorders in children” according to the package insert, and a history of insomnia was common among initiators (45.7% for melatonin and 78.1% for ramelteon), suggesting that these medications were primarily prescribed for insomnia in our study cohort.

This study revealed that BZDs and Z-drugs accounted for a lower proportion of prescriptions of hypnotics. This finding aligns with a broader international trend, as studies from China and several European countries have reported a consistent decline in the prescription of these medications for children and adolescents [22, 23, 26]. This global shift is largely attributable to growing concerns regarding the potential for dependence and other adverse effects associated with the long-term use of these agents in younger populations [23]. Therefore, the low prescription rate of these agents in Japan suggests that clinicians are aligned with the global safety concerns.

### Demographic and clinical characteristics

In Japan, the population <18 years of age is estimated to be approximately 17.86 million. Based on our finding that approximately 0.87% (29 096 patients) of the database population (n = 3 349 453) received a prescription for sleep medication, it can be estimated that approximately 155 000 persons aged <18 years were prescribed hypnotics between 2021 and 2023 in Japan. Our study revealed that, among individuals aged <18 years, the number of patients increased with age. This trend is consistent with that of a study in China, which reported a higher number of patients in older age groups [23]. Furthermore, the proportion of girls increased with age, from 36% in the 0–6 years age group to 60% in the 15–17 years age group. This age- and sex-related difference in the proportion of patients prescribed hypnotics may be partly explained by findings from previous studies reporting that depression and anxiety disorders, which may be associated with insomnia, tend to increase after puberty and throughout adolescence, particularly among girls [33, 34].

This study found a high frequency of psychiatric comorbidities at the initiation of insomnia pharmacotherapy in children and adolescents (ASD, 32.5%; depressive disorders, 23.4%; ADHD, 19.8%; anxiety disorders, 18.2%). These results are consistent with those of a recent US report on youths with insomnia, which also noted substantial comorbidity burdens [22]. The previous Japanese study of hypnotic use reported that the burden of psychiatric comorbidities is higher in children than in adults, with higher prevalences of depression (23.4% vs. 15.3%) and anxiety disorders (18.2% vs. 10.7%) in children [15]. Unlike in adults, where insomnia also reflects stress or lifestyle factors, psychiatric disorders may account for a relatively greater proportion of insomnia in children and adolescents. These findings suggest that pediatric insomnia requires treatment considerations that differ from those in adults and highlight the need for care pathways that combine insomnia management with the coordinated treatment of psychiatric comorbidities.

In our study, the proportion of patients with asthma and atopic dermatitis was higher in the 0–6 years age group than in the other age groups. In contrast, the proportion of patients with these conditions decreased with age in the 12–17 years age group, which may indicate symptom improvement over time [35]. This finding suggests that for younger children, the management of sleep-onset difficulties associated with allergic diseases is a particularly important clinical consideration.

### Prescribing patterns: duration and dosage

A previous study using data from a Japanese claims database to examine the prescription durations of hypnotics in adults, reported continuation rates of 40.2% at 1 month, 29.1% at 3 months, and 10.1% at 1 year [36]. In the present study, the continuation rate was 62.5% at 1 month and 42.6% at 3 months (Figure 1, A), suggesting that the proportion of children who continued hypnotics was higher than that observed in adults. This may reflect that sleep disorders associated with common pediatric comorbidities, such as ASD and ADHD, often require long-term management, indicating a different disease structure than that in adults. When comparing prescription duration by drug type, melatonin had the longest median continuation period of 102 days, with approximately 50% of patients continuing treatment after 30 days. International studies have similarly reported uninterrupted melatonin use in 14–17% of patients even after 1 year. In Japan, melatonin has been revealed to be prescribed to children aged <11 years with ASD and other neurodevelopmental disorders. Since insomnia due to neurodevelopmental conditions such as ASD and ADHD is unlikely to be cured in the short term, long-term treatment is often required, and safer agents such as melatonin appear to be preferentially selected. In contrast, benzodiazepine hypnotics were typically discontinued after a short-term use of approximately 14 days, which likely reflects cautious prescribing practices aimed at minimizing adverse effects, such as dependence.

In this study, we examined original doses of hypnotics and observed median daily values of 1.17 mg/day for melatonin, 6.01 mg/day for ramelteon, and 3.68 mg/day for lemborexant. In Japan, the recommended doses are 1–4, 8, and 2.5–10 mg for melatonin, ramelteon (adults), and lemborexant, respectively. Therefore, the observed medians were at the lower end of the recommended range for melatonin and lemborexant, and at the standard adult dose for ramelteon, indicating a generally conservative dosing pattern at treatment initiation in pediatric care. Although international reports have described higher melatonin doses [25, 26] prescriptions in Japan appear to remain within label-consistent ranges. This dosing profile aligns with clinical caution in youth and facilitates cross-class comparisons using a common equivalence scale. Future studies should examine dose trajectories over time, age- and comorbidity-specific titration practices, and the correlation among dose, effectiveness, and safety in real-world pediatric settings.

### Limitation

This study has some limitations. First, we examined only the patients who initiated prescription of hypnotics and did not capture prevalent users with ongoing use. Furthermore, we only evaluated the prescription patterns of the initiated hypnotics, and we did not assess cases where patients switched from other hypnotics or reinitiated treatment after discontinuation. Therefore, it may be insufficient for a comprehensive evaluation of prevalent insomnia treatment. Future research should investigate practical strategies for changing hypnotics, and the specific factors influencing these decisions. Second, since claims databases do not capture the clinical intent for prescribing and recorded diagnostic codes may not always reflect the actual clinical situation, particularly in pediatric and off-label practice, it may not be possible to accurately ascertain whether patients on hypnotics had insomnia symptoms or symptoms of other sleep disorders. Further research is warranted to better clarify the clinical indications and symptom profiles underlying hypnotic initiation. Third, the follow-up period was limited to 180 days, although some patients may have required long-term therapy. Children with ASD or ADHD may require extended treatment for disease conditions, including insomnia; long-term prescription patterns; and outcomes warrant further investigation. Fourth, we did not investigate dosage form considerations tailored to pediatric needs. As hypnotics other than melatonin are only available in tablet form, it is likely that in clinical practice, tablets are being crushed prior to administration to accommodate younger patients. Fifth, this study represents a substantial proportion of Japanese children and adolescents, and the generalizability of the findings to the Japanese population is considered high. However, due to differences in clinical practices, such as available medications and lifestyle guidance, between Japan and other countries, the results of this study may be difficult to apply to global pediatric care. Finally, since the treatment of pediatric insomnia may affect academic performance and subsequent socioeconomic outcomes, subsequent studies should assess broader social impacts and the overall quality of life.

## Conclusion

The prescription patterns of hypnotics differed by age; in children aged 0–14 years, melatonin and MRAs were the most common, and in adolescents aged 15–17 years, MRAs and DORAs were more frequent. The comorbidities varied with age. Neurodevelopmental disorders, such as ASD and ADHD, were common in younger patients (0–14 years), whereas depressive and anxiety disorders were more commonly observed in older patients (12–17 years). These findings indicate that the clinical approach to the pharmacological treatment of pediatric insomnia in Japan is individualized, considering the patient’s age, developmental stage, and comorbidities. Given the limited existing evidence, further evaluation of the efficacy and safety of sleep medications in Japanese children and adolescents is necessary.

## Supplementary Material

4-Supplementary_material_0225_zsag067(1)

## Data Availability

Individual-level data cannot be shared publicly because of contractual obligations and ethical requirements.
