# Supplementary material for: Prescription patterns of hypnotics for children and adolescents in Japan: a descriptive epidemiologic study using a claims database
Source: Sleep. 2026 Mar 9;49(6):zsag067. doi: 10.1093/sleep/zsag067 (PMC13266551; doi:10.1093/sleep/zsag067)
Supplement: 4-Supplementary_material_0225_zsag067(1) [file 4-supplementary_material_0225_zsag067(1).docx]

**Supplementary Material**

Prescription patterns of hypnotics for children and adolescents in Japan: A descriptive epidemiologic study using a claims database

Sachiko Tanaka-Mizuno ^1.2^, Kenichi Fujimoto ^3^, Kazuo Mishima ^4^, Yukinori Sakata ^3^, Motomu Suga ^5^, Hiroshi Ohashi ^6^, Naoki Kubota ^3^, Michinori Koebis ^3^, Toshiki Fukasawa ^2.7^, Kayoko Mizuno ^2.7^, Mika Ishii ^3^, Margaret Moline ^8^, Koji Kawakami ^2^

^1^ Laboratory of Epidemiology and Prevention, Kobe Pharmaceutical University, Kobe, Japan

^2^ Department of Pharmacoepidemiology, Graduate School of Medicine and Public Health, Kyoto University, Kyoto, Japan

^3^ Eisai Co., Ltd., Tokyo, Japan

^4^ Department of Neuropsychiatry, Akita University Graduate School of Medicine, Akita, Japan.

^5^ Graduate School of Clinical Psychology, Teikyo Heisei University, Tokyo, Japan.

^6^ National Hospital Organization Mie National Hospital, Mie, Japan

^7^ Department of Digital Health and Epidemiology, Graduate School of Medicine and Public Health, Kyoto University, Kyoto, Japan

^8^ Eisai Inc., New Jersey, USA.

**CORRESPONDING AUTHOR**

Sachiko Tanaka-Mizuno, PhD

Professor, Laboratory of Epidemiology and Prevention, Kobe Pharmaceutical University, Kobe, Japan

Address: 4-19-1 Motoyamakitamachi, Higashinada-ku, Kobe, Hyogo 658-8558, Japan

Tel: +81-78-441-7758

E-mail: sachikot@kobepharma-u.ac.jp

# Table S1. Definition of hypnotics

| **Pharmacological class** | **Drug name** | **ATC code** | **Dose equivalence to flunitrazepam (mg/day)** |
| --- | --- | --- | --- |
| Melatonin | Melatonin | N05CH01 | 4 |
| Benzodiazepines  (BZDs) | Estazolam | N05CD04 | 2 |
|  | Flurazepam | N05CD01 | 15 |
|  | Nitrazepam | N05CD02 | 5 |
|  | Triazolam | N05CD05 | 0.25 |
|  | Flunitrazepam | N05CD03 | 1 |
|  | Brotizolam | N05CD09 | 0.25 |
|  | Lormetazepam | N05CD06 | 1 |
|  | Quazepam | N05CD10 | 15 |
|  | Rilmazafone | Defined using ATC code, N05CD, and first 7 digits of YJ code, 1129006. | 2 |
| Z-drugs | Zopiclone | N05CF01 | 7.5 |
|  | Zolpidem | N05CF02 | 10 |
|  | Eszopiclone | N05CF04 | 2.5 |
| Melatonin receptor agonists (MRAs) | Ramelteon | N05CH02 | 8 |
| Dual orexin receptor antagonist (DORAs) | Suvorexant | N05CJ01 | 20 |
|  | Lemborexant | N05CJ02 | 10 |

Abbreviations: ATC code, Anatomical Therapeutic Chemical code; YJ, Yakka Joho code.

# Table S2. Definition of patient and clinical characteristics

| **Patient characteristic** | **Variables** | **Definition** | **Assessment period (days from index date)** |
| --- | --- | --- | --- |
| Background information | Age (years) |  | [0, 0] |
|  | Sex | Male, female | [0, 0] |
| Comorbidity | Insomnia | ICD-10 code: G470 | [−180, 0] |
|  | Allergic rhinitis | ICD-10 code: J30 | [−180, 0] |
|  | ASD | ICD-10 code: F84 | [−180, 0] |
|  | Depression | ICD-10 code: F204, F32, F33, F341 | [−180, 0] |
|  | Asthma | ICD-10 code: J45, J46 | [−180, 0] |
|  | ADHD | ICD-10 code: F90 | [−180, 0] |
|  | Anxiety disorder | ICD-10 code: F40, F41, F930, F940, F064 | [−180, 0] |
|  | Atopic dermatitis | ICD-10 code: L20, L21, L22 | [−180, 0] |
|  | Schizophrenia and psychotropic-related disease | ICD-10 code: F20-F29 | [−180, 0] |
|  | Epilepsy | ICD-10 code: G40 | [−180, 0] |
|  | Intellectual disability | ICD-10 code: F70-F79 | [−180, 0] |
|  | Bipolar disorder | ICD-10 code: F30, F31, F340 | [−180, 0] |
|  | Food allergy | ICD-10 code: T781 | [−180, 0] |
|  | Circadian rhythm sleep disorders | ICD-10 code: G472 | [−180, 0] |
|  | Diabetes | ICD-10 code: E10, E11, E12, E13, E14 | [−180, 0] |
|  | Tic disorder | ICD-10 code: F95 | [−180, 0] |
|  | Anaphylactic Shock | ICD-10 code: T780, T782 | [−180, 0] |
|  | PTSD | ICD-10 code: F431 | [−180, 0] |
|  | Obstructive sleep apnea | ICD-10 code: F951 | [−180, 0] |
| Drug history before the index date | Antipsychotics | ATC code: N05A | [−180, −1] |
|  | Antidepressants | ATC code: N06A | [−180, −1] |
|  | Anxiolytics | ATC code: N05B | [−180, −1] |
|  | ADHD drugs | ATC code: N06BA09, N06BA04, C02AC02, N06BA12 | [−180, −1] |
|  | Antiepileptics | ATC code: N03A | [−180, −1] |
| Concomitant prescribed drug at index date | Antipsychotics | ATC code: N05A | [0, 0] |
|  | Antidepressants | ATC code: N06A | [0, 0] |
|  | Anxiolytics | ATC code: N05B | [0, 0] |
|  | ADHD drugs | ATC code: N06BA09, N06BA04, C02AC02, N06BA12 | [0, 0] |
|  | Antiepileptics | ATC code: N03A | [0, 0] |

Abbreviations: ADHD, Attention-Deficit/Hyperactivity Disorder; ASD: Autism Spectrum Disorder; ATC, Anatomical Therapeutic Chemical; ICD-10, International Classification of Diseases, Tenth Revision; PTSD, Post-Traumatic Stress Disorder.

# Table S3. Baseline characteristics of children and adolescents receiving hypnotic monotherapy by pharmacological class

|  | | |  |  | Melatonin | | BZDs | | Z-drugs | | MRAs | | DORA | | |
| --- | --- | --- | --- | --- | --- | --- | --- | --- | --- | --- | --- | --- | --- | --- | --- |
|  | | |  |  | N=7025 | | N=1297 | | N=2096 | | N=6411 | | N=3907 | | |
|  | | |  |  | n | % | n | % | n | % | n | % | n | % | |
| Sex, girl | | | |  | 3037 | 43.2 | 728 | 56.1 | 1328 | 63.4 | 3603 | 56.2 | 2415 | 61.8 | |
| Age (years old) | | | |  |  |  |  |  |  |  |  |  |  |  | |
|  | Means, SD | | |  | 10.63 | 3.87 | 13.55 | 4.39 | 15.89 | 1.74 | 13.81 | 3.35 | 15.57 | 2.06 |  |
|  | 0–6 years | | |  | 1409 | 20.1 | 167 | 12.9 | 4 | 0.2 | 373 | 5.8 | 21 | 0.5 |  |
|  | 7–11 years | | |  | 2506 | 35.7 | 165 | 12.7 | 56 | 2.7 | 947 | 14.8 | 196 | 5.0 |  |
|  | 12–14 years | | |  | 2398 | 34.1 | 282 | 21.7 | 490 | 23.4 | 2373 | 37.0 | 1009 | 25.8 |  |
|  | 15–17 years | | |  | 712 | 10.1 | 683 | 52.7 | 1546 | 73.8 | 2718 | 42.4 | 2681 | 68.6 |  |
| Comorbidities in Days [−180, 0] | | | | | | | | | | | | | | | |
|  | | Insomnia | |  | 3210 | 45.7 | 791 | 61.0 | 1668 | 79.6 | 5004 | 78.1 | 3310 | 84.7 |  |
|  | | Allergic rhinitis | |  | 3508 | 49.9 | 493 | 38.0 | 673 | 32.1 | 2533 | 39.5 | 1297 | 33.2 |  |
|  | | ASD | |  | 3781 | 53.8 | 247 | 19.0 | 224 | 10.7 | 1764 | 27.5 | 775 | 19.8 |  |
|  | | Depression | |  | 668 | 9.5 | 388 | 29.9 | 792 | 37.8 | 1387 | 21.6 | 1572 | 40.2 |  |
|  | | Asthma | |  | 2028 | 28.9 | 249 | 19.2 | 245 | 11.7 | 1170 | 18.2 | 476 | 12.2 |  |
|  | | ADHD | |  | 2160 | 30.7 | 152 | 11.7 | 178 | 8.5 | 1120 | 17.5 | 519 | 13.3 |  |
|  | | Anxiety disorder | |  | 926 | 13.2 | 245 | 18.9 | 501 | 23.9 | 1085 | 16.9 | 1012 | 25.9 |  |
|  | | Atopic dermatitis | |  | 1218 | 17.3 | 186 | 14.3 | 242 | 11.5 | 845 | 13.2 | 429 | 11.0 |  |
|  | | Schizophrenia and psychotropic-related disease | |  | 606 | 8.6 | 294 | 22.7 | 322 | 15.4 | 711 | 11.1 | 791 | 20.2 |  |
|  | | Epilepsy | |  | 560 | 8.0 | 150 | 11.6 | 95 | 4.5 | 472 | 7.4 | 185 | 4.7 |  |
|  | | Intellectual disability | |  | 792 | 11.3 | 94 | 7.2 | 41 | 2.0 | 371 | 5.8 | 123 | 3.1 |  |
|  | | Bipolar disorder | |  | 144 | 2.0 | 110 | 8.5 | 150 | 7.2 | 281 | 4.4 | 329 | 8.4 |  |
|  | | Food allergy | |  | 322 | 4.6 | 18 | 1.4 | 32 | 1.5 | 201 | 3.1 | 74 | 1.9 |  |
|  | | Circadian rhythm sleep disorders | |  | 208 | 3.0 | 2 | 0.2 | 11 | 0.5 | 195 | 3.0 | 37 | 0.9 |  |
|  | | Diabetes | |  | 57 | 0.8 | 28 | 2.2 | 50 | 2.4 | 95 | 1.5 | 60 | 1.5 |  |
|  | | Tic disorder | |  | 88 | 1.3 | 5 | 0.4 | 5 | 0.2 | 31 | 0.5 | 18 | 0.5 |  |
|  | | Anaphylactic shock | |  | 51 | 0.7 | 9 | 0.7 | 16 | 0.8 | 35 | 0.5 | 20 | 0.5 |  |
|  | | PTSD | |  | 29 | 0.4 | 10 | 0.8 | 16 | 0.8 | 32 | 0.5 | 31 | 0.8 |  |
|  | | Obstructive sleep apnea | |  | 47 | 0.7 | 8 | 0.6 | 8 | 0.4 | 42 | 0.7 | 13 | 0.3 |  |
|  | | Restless legs syndrome | |  | 25 | 0.4 | 3 | 0.2 | 5 | 0.2 | 15 | 0.2 | 9 | 0.2 |  |
|  | | Orthostatic hypotension | |  | 9 | 0.1 | 0 | 0.0 | 0 | 0.0 | 4 | 0.1 | 2 | 0.1 |  |
| Psychotropic prescribed in Days [−180, −1] | | | | | | | | | | | | | | |  |
|  | Antipsychotics | | |  | 1488 | 21.2 | 277 | 21.4 | 354 | 16.9 | 1117 | 17.4 | 897 | 23.0 | |
|  | Antidepressants | | |  | 337 | 4.8 | 177 | 13.6 | 332 | 15.8 | 577 | 9.0 | 725 | 18.6 | |
|  | Anxiolytics | | |  | 503 | 7.2 | 281 | 21.7 | 440 | 21.0 | 742 | 11.6 | 711 | 18.2 | |
|  | ADHD drugs | | |  | 1241 | 17.7 | 81 | 6.2 | 84 | 4.0 | 668 | 10.4 | 285 | 7.3 | |
|  | Antiepileptics | | |  | 537 | 7.6 | 146 | 11.3 | 127 | 6.1 | 480 | 7.5 | 268 | 6.9 | |
| Psychotropic prescribed in Day [0] | | | | | | | | | | | | | | | |
|  | Antipsychotics | | |  | 1319 | 18.8 | 335 | 25.8 | 353 | 16.8 | 1050 | 16.4 | 872 | 22.3 | |
|  | Antidepressants | | |  | 259 | 3.7 | 202 | 15.6 | 415 | 19.8 | 507 | 7.9 | 802 | 20.5 | |
|  | Anxiolytics | | |  | 226 | 3.2 | 293 | 22.6 | 489 | 23.3 | 457 | 7.1 | 570 | 14.6 | |
|  | ADHD drugs | | |  | 1088 | 15.5 | 60 | 4.6 | 72 | 3.4 | 549 | 8.6 | 225 | 5.8 | |
|  | Antiepileptics | | |  | 366 | 5.2 | 106 | 8.2 | 90 | 4.3 | 284 | 4.4 | 186 | 4.8 | |

ADHD, Attention-Deficit/Hyperactivity Disorder; ASD, Autism Spectrum Disorder; BZD, Benzodiazepine; DORA: Dual orexin receptor antagonist; MRAs, Melatonin receptor agonists; PTSD, Post-Traumatic Stress Disorder; SD, Standard Deviation.

# Figure S1. Patients flow


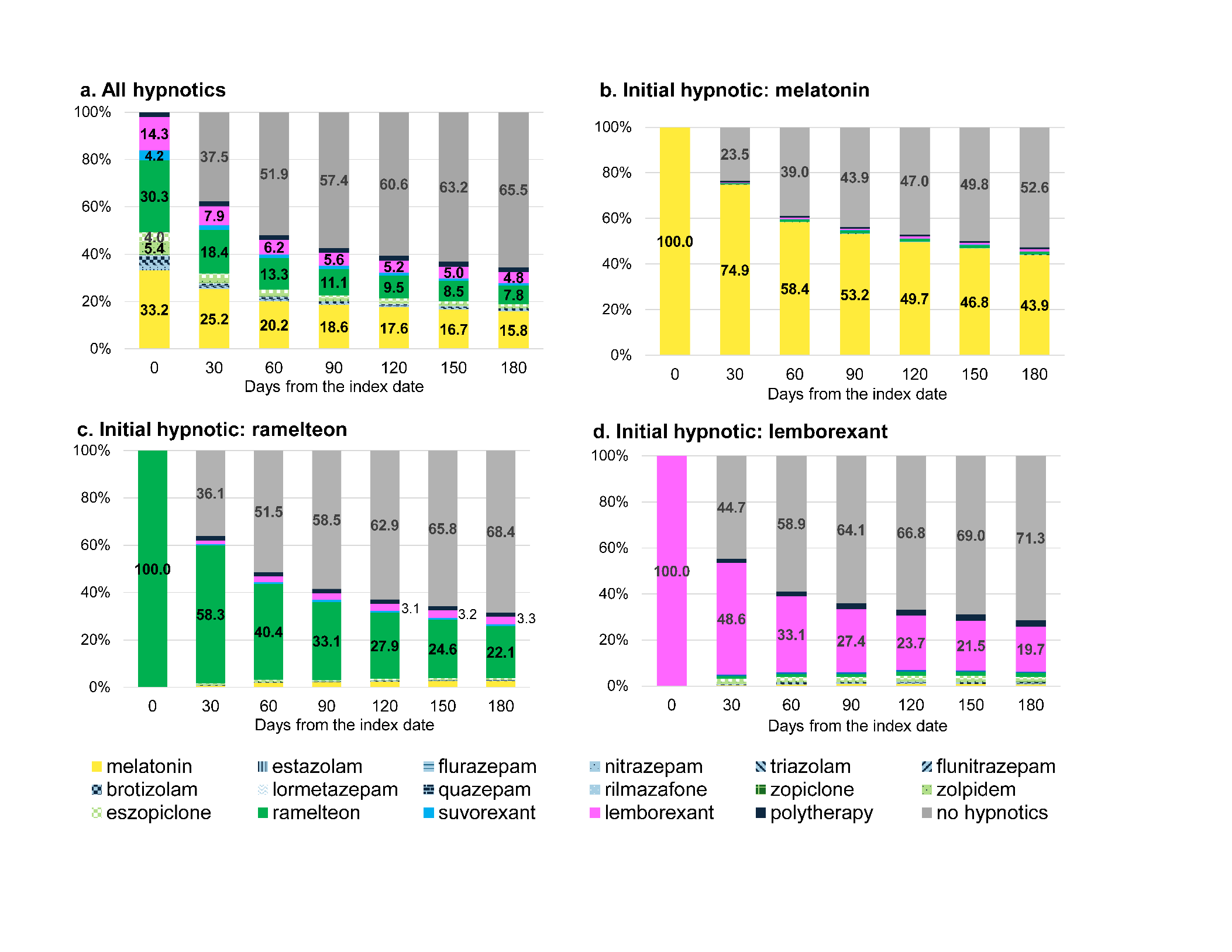


#
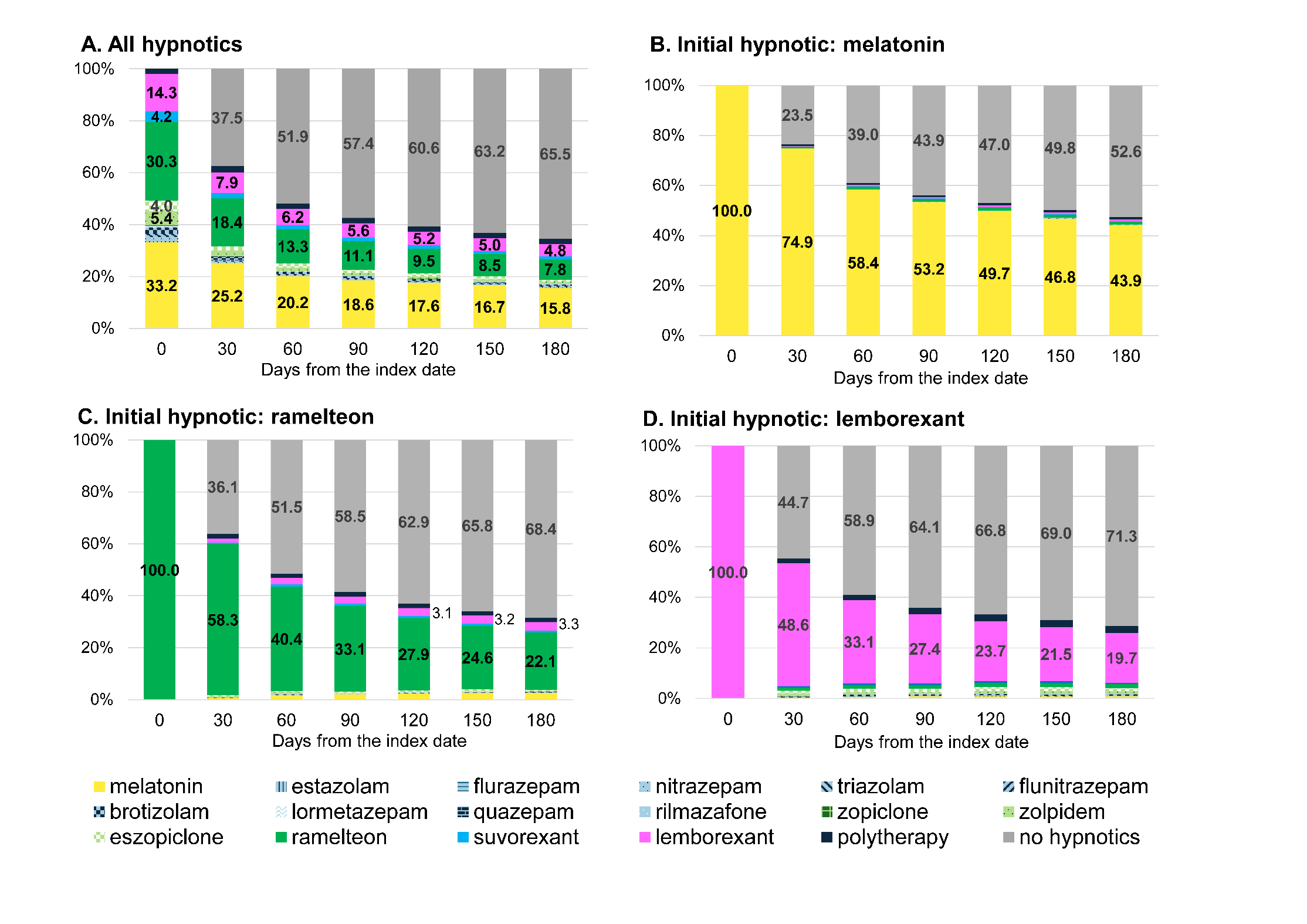
Figure S2. Longitudinal changes in insomnia medication patterns by initial hypnotics

Stacked bar charts show the proportions of patients at each time point who were receiving each hypnotic, polytherapy (≥2 hypnotics), or no hypnotics.
Panels show (A) all hypnotics, (B) initial prescription: melatonin, (C) initial prescription: ramelteon, and (D) initial prescription: lemborexant.

A single legend is shown for all panels. Values of proportion <3% are not labeled in the figure.
